# Nomophobia Among Adolescents in Kyrgyzstan: Scores, Dimensions, and Associations With Smartphone‐Use Patterns

**DOI:** 10.1002/hsr2.73501

**Published:** 2026-11-10

**Authors:** Maksutova Begaiym, Ergeshova Seyil Baktybekovna, Bedir Eroğlu

**Affiliations:** ^1^ Department Advertising and Public Relations of Kyrgyz National University Bishkek Kyrgyzstan; ^2^ Osh State University Osh Kyrgyzstan; ^3^ Kyrgyzs‐Turkish Manas University Bishkek Kyrgyzstan

**Keywords:** adolescents, digital behavior, Kyrgyzstan, nomophobia, smartphone use

## Abstract

**Background and Aims:**

This study examines overall and dimension‐specific nomophobia scores among adolescents in Kyrgyzstan and analyzes their associations with demographic characteristics and smartphone‐use patterns.

**Methods:**

A cross‐sectional, descriptive, and correlational study was conducted in Bishkek, Kyrgyzstan, between June 2 and June 25, 2026. Data were collected from 400 adolescents aged 13–17 years using a culturally adapted Russian‐language 5‐point version of the Nomophobia Questionnaire. Data were analyzed using descriptive statistics, independent‐samples *t*‐tests, one‐way ANOVA, Pearson correlation, and hierarchical multiple regression.

**Results:**

The overall item‐level nomophobia score was 2.62 (SD = 0.81). Among the four dimensions, Not Being Able to Communicate had the highest mean score, whereas Losing Connectedness had the lowest. Female adolescents reported significantly higher observed nomophobia scores than males; however, measurement invariance across gender was not examined, and this comparison should therefore be interpreted cautiously. No significant differences were found according to age, grade level, or parental attitudes toward internet and social media use. Significant omnibus ANOVA results were observed for both years of smartphone use and daily smartphone‐use duration. However, none of the Tukey‐adjusted pairwise comparisons for years of smartphone use reached statistical significance; therefore, no differences between specific smartphone‐ownership‐duration groups were identified. Tukey‐adjusted post hoc comparisons for daily smartphone use showed that adolescents using smartphones for 4–6 h and 7 h or more per day had higher nomophobia scores than those using them for 1–3 h per day. The final regression model was statistically significant and explained 19.3% of the variance; in this model, the 1–3‐h category was associated with lower nomophobia scores than the 4–6‐h reference category.

**Conclusion:**

Nomophobia among adolescents in Kyrgyzstan was primarily associated with communication‐related concerns, gender, and current daily smartphone‐use patterns. Female gender and more intensive daily smartphone use showed stronger associations with nomophobia than age, grade level, parental attitudes, or duration of smartphone ownership. Given the cross‐sectional design, convenience sampling, self‐report data, and limited explained variance, the findings should be interpreted as associations rather than causal or clinical evidence.

## Abstract (highlights)

**Summary:**

- **What is known**
- Nomophobia is associated with communication concerns and intensive smartphone use, but evidence concerning adolescents in Central Asia remains limited.
- **What is new**
- Communication‐related concerns represented the dimension with the highest observed mean of nomophobia, while female gender and daily smartphone‐use patterns were more consistently associated with nomophobia than age, grade level, parental attitudes, or years of smartphone use.

**Implications:**

Future research should examine psychological, interpersonal, and family‐related factors alongside smartphone‐use patterns before drawing clinical, educational, or policy implications.

## 1 Introduction

Nomophobia has attracted increasing scholarly attention as a smartphone‐related form of anxiety involving distress or discomfort when access to mobile communication, information, and digital services is interrupted [1, 2]. Although nomophobia is not recognized as a clinical diagnosis, recent systematic evidence indicates that it is widely reported across different populations and commonly occurs at mild to moderate levels [3]. These concerns may be particularly relevant during adolescence, as smartphones have become increasingly embedded in young people's communication, peer relationships, family interactions, and everyday routines. Recent adolescent‐focused research has also associated nomophobia with problematic social media use, social anxiety, and broader psychosocial difficulties, underscoring its relevance to adolescent digital well‐being and mental health [4]. However, much of the existing evidence has been derived from university students and populations outside Central Asia, while research focusing specifically on adolescents in this region remains limited. This gap is important because adolescents' experiences of smartphone‐related anxiety may be shaped by developmental needs for social connection, family relationships, patterns of digital engagement, and the broader sociocultural environment.

The widespread adoption of smartphones has transformed how individuals communicate, access information, and participate in everyday life. Beyond their practical functions, smartphones have become embedded in adolescents' peer relationships, social interactions, and daily routines [5]. Although these technologies provide substantial communicative and informational benefits, intensive or problematic smartphone use has also been associated with poorer psychological well‐being, reduced quality of life, and other adverse mental health outcomes among children and adolescents [6, 7]. Within this broader digital environment, nomophobia reflects concerns not only about losing access to the device itself but also about disruptions in communication, information access, and social connectedness [8, 9].

Social Cognitive Theory provides a useful framework for interpreting nomophobia as a response shaped by reciprocal interactions among individual characteristics, behavior, and the digital environment [10]. Repeated exposure to messages, notifications, and socially rewarding feedback may reinforce expectations of continuous accessibility and habitual smartphone engagement. Evidence indicating that heightened sensitivity to social rewards is associated with problematic smartphone use among adolescents lends support to this interpretation [11]. Over time, these reinforcement processes may increase adolescents' sensitivity to interruptions in smartphone access. However, because social reward sensitivity and reinforcement mechanisms were not directly measured, Social Cognitive Theory is used only as an interpretive framework in the present study.

Nomophobia is widely recognized as a multidimensional phenomenon encompassing concerns related to communication, information access, social connectedness, and everyday convenience [2]. Although it may occur across age groups, adolescence represents a particularly sensitive developmental period because of identity formation, peer relationships, emotional regulation, and increasing reliance on digital communication. Adolescent studies have associated higher nomophobia levels with intensive smartphone use, social media engagement, social anxiety, loneliness, stress, depression, and poorer psychological well‐being [1, 12, 13]; Maghaireh et al., 2025 [4];). Research has also indicated that female adolescents and those reporting longer daily smartphone use may experience higher nomophobia levels, although these associations vary across samples and cultural settings [3, 13, 14]. Collectively, these findings suggest that nomophobia should be understood within developmental, behavioral, and sociocultural contexts rather than solely as an individual psychological characteristic.

Cultural context may contribute to how individuals experience mobile connectivity and smartphone‐related anxiety. Previous research has associated collectivist orientations with nomophobia, suggesting that culturally shaped expectations concerning interpersonal availability and connectedness may influence responses to mobile disconnection [15]. Cross‐national evidence has also demonstrated substantial variation in the prevalence and severity of nomophobia across countries, indicating that experiences of smartphone‐related anxiety may differ according to broader social and digital environments [3, 16]. More broadly, cultural dimensions may influence how individuals adopt, use, and assign social meaning to digital technologies [17]. Nevertheless, technology acceptance, problematic smartphone use, and nomophobia remain conceptually distinct constructs. Cultural context is therefore used only as an interpretive background in the present study, as cultural orientations were not directly measured.

Despite increasing digitalization, adolescents in Kyrgyzstan remain underrepresented in nomophobia research. Existing evidence has concentrated predominantly on university students, reflecting a broader tendency within the nomophobia literature to examine higher‐education populations rather than younger adolescents [1, 18]. Consequently, limited evidence is available regarding the observed mean patterns across the nomophobia dimensions among secondary‐school adolescents in Kyrgyzstan or the demographic and smartphone‐use factors associated with their overall nomophobia scores.

Kyrgyzstan represents a rapidly digitalizing context characterized by extensive internet and mobile connectivity. According to the International Telecommunication Union, approximately 92% of individuals in Kyrgyzstan used the internet in 2024, while active mobile‐broadband subscriptions reached 92.3 per 100 inhabitants [19]. These indicators demonstrate that mobile and internet technologies have become widely accessible within the country. At the same time, Freedom House [20] documents the expanding role of online communication and internet access in Kyrgyzstan. International public health evidence further indicates that intensive and problematic social media use has become an increasingly relevant concern for adolescent well‐being as digital engagement expands [21]. However, evidence concerning the psychological and communication‐related implications of intensive smartphone use among adolescents in Kyrgyzstan remains limited, underscoring the need for context‐specific research on adolescent nomophobia.

Emerging evidence from Kyrgyzstan has begun to document digital risks and smartphone‐related psychosocial experiences among children and adolescents. Previous research has examined cyberbullying among children in Kyrgyzstan, demonstrating the growing relevance of digitally mediated risks within this population [22]. More recently, nomophobia was found to be positively associated with social isolation among Kyrgyz adolescents [23]. However, that study focused specifically on the relationship between overall nomophobia and social isolation. The present study extends this local evidence by examining the observed mean patterns and interrelationships of the four nomophobia dimensions and by evaluating the associations of demographic characteristics, smartphone‐use patterns, and parental attitudes with overall nomophobia scores.

Recent studies from Central Asia have primarily focused on university students. For example [24]) examined nomophobia among university students in Kyrgyzstan and Azerbaijan, while studies conducted in Kazakhstan explored its associations with psychological well‐being, academic outcomes, and technology‐related behaviors [25, 26]. Similar patterns have also been reported in broader international research, where university students remain the most frequently investigated population in nomophobia studies [3, 18]. However, evidence focusing specifically on secondary‐school adolescents remains limited, particularly in Central Asia.

Building on these gaps, the present study examines overall and dimension‐specific nomophobia scores among adolescents in Kyrgyzstan and evaluates their associations with demographic characteristics, smartphone‐use patterns, and parental attitudes. By focusing on an underrepresented adolescent population in Central Asia, the study extends existing evidence beyond predominantly university‐based samples and contributes context‐specific findings that may inform the future development and evaluation of school‐based interventions and adolescent digital well‐being initiatives.

To address this objective, the study is guided by the following research questions.

RQ1: What are the overall and dimension‐specific nomophobia scores among adolescents in Kyrgyzstan?

RQ2: How are the dimensions of nomophobia related to one another among adolescents in Kyrgyzstan?

RQ3: Do adolescents' nomophobia scores differ according to demographic characteristics and smartphone‐use patterns?

RQ4: To what extent are gender, age, years of smartphone use, daily smartphone use, and parental attitudes toward internet and social media use associated with adolescents' overall nomophobia scores, and how much variance do they account for?

## 2 Materials and Methods

### 2.1 Research Design

This study employed a cross‐sectional, descriptive, and correlational design to examine nomophobia scores among adolescents in Kyrgyzstan and their associations with demographic characteristics and smartphone‐use patterns.

### 2.2 Participants, Sampling Procedures, and Ethical Considerations

Data collection was conducted between June 2 and June 25, 2026, among adolescents aged 13–17 years in Bishkek, Kyrgyzstan. Recruitment was conducted at the individual adolescent level through a family‐mediated convenience‐sampling procedure rather than through formally selected schools or classroom‐based recruitment. Attendance at a public co‐educational school in central Bishkek was used as an eligibility criterion, but schools themselves were not treated as sampling or recruitment units. School names and school affiliations were not recorded; consequently, the number of schools represented in the final sample could not be determined.

Parents of potentially eligible adolescents were approached through the researchers' personal and local community contacts and were informed about the purpose and voluntary nature of the study. Each parent was asked to provide the questionnaire package voluntarily to one adolescent who met the stated eligibility criteria. Adolescents completed the questionnaires outside formal classroom settings and were instructed to respond independently. Completed questionnaires were returned through their parents. Approximately 450 adolescents were approached through this procedure, corresponding to the 450 questionnaire forms distributed.

To protect participants' anonymity, the questionnaires did not request names, school names, student identification numbers, or other directly identifying information. Parents were informed about the purpose of the study and were asked not to assist, influence, or otherwise intervene while the adolescents completed the questionnaires so that responses could be provided independently. Teachers and school personnel were not involved in recruitment, questionnaire administration, or the return of completed questionnaires and did not have access to the responses. However, completed questionnaires were not returned in sealed envelopes. Therefore, although responses were anonymous to the researchers, parental access to individual responses before the questionnaires were returned could not be fully prevented or verified.

Eligibility criteria required participants to be between 13 and 17 years of age, reside in Bishkek, attend a public co‐educational school, have regular internet access, and use a personal smartphone. Adolescents who did not meet these criteria were not included in the study.

A total of 450 questionnaire forms were distributed. Of these, 400 complete and eligible questionnaires were retained for analysis, representing 88.9% of the distributed forms. The remaining 50 forms were not included because they were either not returned or contained substantial missing responses. Because separate counts were not recorded for non‐returned and incomplete questionnaires, the 88.9% figure should be interpreted as the proportion of distributed questionnaires retained for analysis rather than as a conventional survey response rate. No item‐level missing data remained in the final analytical dataset, and no statistical imputation was performed. The final analytical sample therefore consisted of 400 adolescents.

An a priori power analysis was originally conducted using G*Power 3.1 [27] for multiple linear regression, with 10 predictors, a medium effect size (f^2^ = 0.15), an alpha level of 0.05, and statistical power of 0.95. This calculation yielded a minimum required sample size of 172. The final regression model contains 11 predictor coefficients because years of smartphone use, daily smartphone‐use duration, and parental attitudes were dummy‐coded, contributing three coefficients each, in addition to one coefficient for gender and one for age. To align the sample‐size assessment with the final model specification, the required sample size was recalculated using 11 predictors and the same assumptions, yielding a minimum of 178 participants. This recalculation was conducted after data collection as a check against the final model. The final sample of 400 adolescents exceeded this minimum.

Ethical approval for the study was obtained from the Ethics Committee of Osh State University, Kyrgyzstan (Protocol No. 6, June 1, 2026). Written informed consent was obtained from the parents or legal guardians of all participating adolescents, and written assent was obtained from the adolescents before participation. Participation was voluntary, and no names, school identifiers, student identification numbers, or other directly identifying information were collected. All procedures were conducted in accordance with the Declaration of Helsinki.

### 2.3 Measurement

The data collection instrument consisted of three sections: (1) demographic information, (2) smartphone‐use patterns, and (3) the Nomophobia Questionnaire (NMP‐Q). Demographic variables included gender, age, and grade level. The second section assessed smartphone‐use patterns, including years of smartphone use, daily use duration, parental attitudes toward internet and social media use, preferred communication channels, and purposes of smartphone use.

### 2.4 Nomophobia Questionnaire (NMP‐Q)

The Nomophobia Questionnaire (NMP‐Q), developed by Yildirim and Correia [2], was used to assess participants' nomophobia. The original instrument consists of 20 items rated on a 7‐point Likert‐type agreement scale. The present study used an existing Russian‐language 5‐point version of the NMP‐Q obtained from Ayhan, who had previously administered this version to adolescents in Kyrgyzstan [23]. The obtained version used a frequency‐based response format ranging from 1 (never) to 5 (always). The present authors did not independently change the number of response categories or develop a new Russian translation. The use of five response categories was considered appropriate for providing adolescents aged 13–17 years with a relatively simple response structure. The instrument comprises four dimensions: Not Being Able to Communicate, Not Being Able to Access Information, Losing Connectedness, and Giving Up Convenience. Overall and dimension scores were calculated as item‐level means ranging from 1 to 5, with higher scores indicating greater nomophobia. Because both the number and wording of the response categories differed from the original 7‐point agreement format, the severity thresholds proposed for the original NMP‐Q were not applied.

### 2.5 Adaptation Process and Construct Examination

Permission to use and adapt the original NMP‐Q developed by Yildirim and Correia [2] was obtained from the scale developers through email correspondence. For the present study, the authors obtained the Russian‐language 5‐point NMP‐Q and the accompanying questionnaire materials directly from Ayhan through email correspondence and used them with permission [23]. This version had previously been administered to adolescents in Kyrgyzstan. However, its previous use was not regarded as sufficient evidence of validity for the current sample.

Before data collection, the present authors conducted a back‐translation procedure to examine the linguistic and conceptual equivalence of the obtained Russian‐language version. The Russian items were translated back into English and compared with the corresponding items of the original English‐language NMP‐Q. The authors reviewed the original and back‐translated versions for consistency in meaning, conceptual equivalence, and suitability for adolescent respondents. No substantive discrepancies requiring modification of the Russian items were identified. The item wording was also reviewed qualitatively for clarity and cultural suitability; however, a quantitative content‐validity index was not calculated. This qualitative review was conducted by the authors rather than by a formally constituted independent expert panel, and formal individual cognitive interviews were not conducted.

The Russian‐language version was subsequently pilot‐tested with 30 adolescents aged 13–17 years to assess item clarity, comprehensibility, and cultural suitability. No item modifications were considered necessary following the pilot study. The adolescents who participated in the pilot study were not included in the final analytical sample of 400 participants. Preliminary evidence regarding the factor structure and internal consistency of the instrument was subsequently examined in the current sample through EFA, CFA, Cronbach's alpha, and McDonald's omega rather than being assumed solely on the basis of its previous use. However, these procedures do not establish full linguistic or measurement equivalence with the original seven‐point NMP‐Q.

An exploratory factor analysis (EFA) using principal axis factoring with Direct Oblimin rotation was conducted to examine the factor structure of the Russian‐language 5‐point NMP‐Q. Because the original NMP‐Q specifies four theoretically related dimensions, a four‐factor solution was retained a priori. The Kaiser–Meyer–Olkin measure indicated good sampling adequacy (KMO = 0.903), and Bartlett's test of sphericity was statistically significant, χ^2^(190) = 2980.21, *p* < 0.001. The four‐factor solution accounted for 44.73% of the total variance, with absolute primary factor loadings ranging from 0.28 to 0.86. The solution was broadly consistent with the original four‐dimensional structure, although some item‐level deviations and cross‐loadings were observed. Accordingly, the EFA findings were interpreted as preliminary exploratory evidence rather than as confirmation of the factor structure. The complete rotated EFA pattern matrix, including loadings across all four factors, is presented in Supporting Information: Table S3.

To obtain additional preliminary evidence regarding the factor structure of the adapted 5‐point NMP‐Q, a confirmatory factor analysis (CFA) was conducted based on the original four‐factor structure proposed by Yildirim and Correia [2]. The model yielded mixed fit indices: χ^2^/df = 3.64, CFI = 0.868, TLI = 0.847, RMSEA = 0.075, and SRMR = 0.058. Although the RMSEA and SRMR values were within acceptable ranges, the CFI and TLI values were below the commonly recommended threshold of 0.90. Taken together, the CFA findings provided only preliminary and partial support for the original four‐factor structure of the adapted instrument. The standardized factor loadings ranged from 0.390 to 0.836, while the correlations among the four latent factors ranged from 0.488 to 0.840. The complete standardized CFA factor loadings and latent‐factor correlations are presented in Supporting Information: Tables S1 and S2, respectively. In particular, the CFI and TLI values did not support a conclusion that the four‐factor structure had been strongly confirmed. Moreover, because the EFA and CFA were conducted using the same sample, the factor structure was not independently cross‐validated; further validation with an independent sample is therefore required to establish the stability of the Russian‐language 5‐point version.

### 2.6 Reliability Analysis

In the present study, the adapted 5‐point NMP‐Q demonstrated good overall internal consistency (Cronbach's α = 0.898; McDonald's ω = 0.893). Internal consistency estimates for the four dimensions were as follows: Not Being Able to Communicate (α = 0.884; ω = 0.885), Losing Connectedness (α = 0.767; ω = 0.768), Not Being Able to Access Information (α = 0.701; ω = 0.705), and Giving Up Convenience (α = 0.686; ω = 0.692). Thus, reliability was good for the overall scale and ranged from marginal to good across the four dimensions. The comparatively lower coefficients for Giving Up Convenience indicate that findings for this dimension should be interpreted with some caution.

### 2.7 Data Analysis

The data analysis process was conducted using IBM SPSS Statistics 23.0 and R. Prior to analysis, the dataset was screened for missing values and outliers. Normality assumptions were evaluated using skewness and kurtosis statistics, and all variables were found to fall within the acceptable range of ±2.

To examine the latent factor structure of the Russian‐language 5‐point NMP‐Q in the current sample, an exploratory factor analysis (EFA) using principal axis factoring with Direct Oblimin rotation was conducted. A four‐factor solution was specified a priori based on the original theoretical structure of the NMP‐Q. Confirmatory factor analysis (CFA) was subsequently conducted in R using the lavaan package [28] to evaluate the fit of the original four‐factor measurement model. Although the NMP‐Q items were ordinal, each item had five ordered response categories and the observed skewness and kurtosis values were within ±2. Therefore, the indicators were treated as approximately continuous, and model parameters were estimated using maximum likelihood estimation. This analytical decision should be considered when interpreting the CFA findings. The original four‐factor measurement model was tested without cross‐loadings or correlated residuals. No post hoc modifications were made to the model, and modification indices were not used to respecify the model. Internal consistency reliability was assessed using both Cronbach's alpha and McDonald's omega coefficients.

Descriptive statistics, including frequencies, percentages, means, and standard deviations, were calculated to summarize participants' demographic characteristics, smartphone‐use patterns, and nomophobia scores. Independent‐samples *t*‐tests and one‐way analysis of variance (ANOVA) were conducted to examine differences in nomophobia scores across demographic characteristics and smartphone‐use patterns. Homogeneity of variance was assessed using Levene's test, and Welch's ANOVA was used as a sensitivity analysis when this assumption was violated. Effect sizes were reported alongside the group‐comparison tests. Cohen's d was calculated for the gender comparison, and partial eta squared (ηp^2^) was calculated for the one‐way ANOVA results. A 95% confidence interval was also calculated for the mean gender difference. Pearson correlation analysis was used to assess the relationships among the four dimensions of nomophobia.

In addition, hierarchical multiple regression analysis was performed to examine the associations of gender, age, years of smartphone use, daily smartphone use, and parental attitudes toward internet and social media use with overall nomophobia scores and the variance accounted for by successive blocks of variables. Given the cross‐sectional design, the regression coefficients were interpreted as conditional associations and explained variance rather than as evidence of causal or temporal prediction. The order of entry was specified to distinguish the contribution of basic demographic and developmental characteristics, accumulated smartphone‐use experience, and more proximal behavioral and family‐contextual factors. This ordering was informed by a developmental and Social Cognitive perspective: relatively stable demographic and developmental characteristics were controlled first, accumulated exposure to smartphone use was entered second, and current behavioral engagement and family‐contextual factors were entered last as more proximal correlates of nomophobia. Gender and age were entered simultaneously in the first step as basic demographic and developmental covariates. Years of smartphone use was entered in the second step to represent accumulated smartphone‐use experience. Daily smartphone use and parental attitudes were entered in the third step because they represent current behavioral engagement and the family context surrounding adolescents' digital‐media use. Grade level was not entered together with age because these variables represent closely overlapping indicators of adolescents' developmental and educational position; age was retained as the more direct developmental covariate. Parental attitudes were retained despite the nonsignificant group comparison because of their theoretical relevance to adolescent digital behavior and parental mediation. Gender was entered as a binary predictor, age was entered as a continuous variable in years, and years of smartphone use, daily smartphone use, and parental attitudes were dummy‐coded. For each categorical predictor, the category containing the largest number of participants was selected as the reference group to support stable coefficient estimation and avoid using relatively small groups as the baseline. Accordingly, the reference categories were more than 5 years of smartphone use (n = 224), four to 6 h of daily smartphone use (n = 166), and parents who warned adolescents about online risks but did not restrict their internet or social media use (n = 239). Although the four‐to‐6‐h category does not represent the lowest level of daily use, it was retained as the reference because it was the largest daily‐use group and therefore provided the most stable baseline for the regression coefficients. To facilitate substantive interpretation, the regression findings were considered together with the group means and Tukey‐adjusted comparisons reported in Table 5. Because the group reporting less than 1 h of daily smartphone use contained only 13 participants, a sensitivity analysis was also conducted by combining this group with the 1–3‐h group to create a 3‐h‐or‐less category and re‐estimating the final regression model.

Prior to interpreting the hierarchical regression models, the assumptions of linearity, residual normality, homoscedasticity, independence of errors, multicollinearity, and the absence of unduly influential observations were examined. Residual plots and the normal probability plot did not indicate substantial violations of linearity, homoscedasticity, or residual normality. Tolerance and variance inflation factor values indicated no problematic multicollinearity among the predictors. The Durbin–Watson statistic was 1.929, supporting the independence of residuals, and Cook's distance values did not indicate that the model was unduly influenced by any single observation. In all analyses, the level of statistical significance was set at *p* < 0.05.

## 3 Results

### 3.1 Participants' Demographic Characteristics and Smartphone‐Use Patterns

A total of 400 adolescents participated in the study. The sample was nearly balanced in terms of gender, comprising 196 males (49.0%) and 204 females (51.0%).

Participants ranged in age from 13 to 17 years. The largest age group was 13‐year‐olds (24.8%), followed by 16‐year‐olds (22.8%) and 14‐year‐olds (21.8%).

Regarding grade level, the largest proportion of participants were enrolled in 7th grade (40.8%), followed by 10th grade (23.0%) and 11th grade (18.5%). Smaller proportions were observed for 9th grade (11.3%) and 8th grade (6.5%) (Table 1).

**Table 1:** Demographic characteristics of adolescents (*N* = 400).

Variable	Category	*n*	%
Gender	Male	196	49.0
	Female	204	51.0
Age	13 years old	99	24.8
	14 years old	87	21.8
	15 years old	46	11.5
	16 years old	91	22.8
	17 years old	77	19.3
Grade level	7th grade	163	40.8
	8th grade	26	6.5
	9th grade	45	11.3
	10th grade	92	23.0
	11th grade	74	18.5

More than half of the participants (56.0%) reported having used smartphones for more than 5 years, while 25.3% had used them for 4 to 5 years.

Regarding daily smartphone use, the largest group reported using smartphones for 4–6 h per day (41.5%), followed by those who used them for 7 h or more (26.8%). Only 3.3% reported using smartphones for less than 1 h per day.

Most participants (59.8%) reported that their parents warned them about the potential risks of internet and social media use but did not impose restrictions. A smaller proportion reported active parental monitoring and limitation (9.8%), while 22.5% indicated that their parents did not intervene.

Instagram and WhatsApp were the most commonly reported platforms (67.3% each), followed by TikTok (64.8%), YouTube (50.2%), and Telegram (46.5%).

Face‐to‐face interaction was the most frequently reported communication channel with friends (73.0%), followed by WhatsApp (15.5%) and Instagram (9.0%).

When asked how they felt without their smartphones, 40.8% reported feeling comfortable, 36.5% reported feeling slightly anxious, 16.3% reported feeling anxious, and 6.5% reported feeling panicked.

Smartphones were most commonly used for social media (87.5%) and entertainment (66.5%). Educational use was reported by 55.0% of participants, while 24.5% reported using smartphones for news or discussion platforms (Table 2).

**Table 2:** Smartphone‐use patterns (*N* = 400).

Variable	Category	n	%
Years of smartphone use	1 year or less	22	5.5
	2–3 years	53	13.3
	4–5 years	101	25.3
	More than 5 years	224	56.0
Daily smartphone use	Less than 1 h	13	3.3
	1–3 h	114	28.5
	4–6 h	166	41.5
	7+ hours	107	26.8
Parental attitude toward internet/social media use	Actively monitor and limit	39	9.8
	Warn but do not limit	239	59.8
	No intervention	90	22.5
	Never discussed	32	8.0
Most used social media platforms	TikTok	259	64.8
	Instagram	269	67.3
	YouTube	201	50.2
	WhatsApp	269	67.3
	Telegram	186	46.5
Main communication channel with friends	In person	292	73.0
	WhatsApp	62	15.5
	Instagram	36	9.0
	Discord	9	2.3
	Telegram	1	0.3
Feeling when without smartphone	Comfortable	163	40.8
	Slightly anxious	146	36.5
	Anxious	65	16.3
	Panicked	26	6.5
Main purposes of smartphone use	Social media	350	87.5
	News/discussion platforms	98	24.5
	Entertainment	266	66.5
	Education	220	55.0

*Note:* Percentages were calculated based on valid responses (*N* = 400). Multiple responses were permitted for social media platform use and purposes of smartphone use.

### 3.2 Descriptive Findings on Nomophobia Among Adolescents

Table 3 presents the descriptive statistics for the overall nomophobia score and its four dimensions. Among the dimensions, Not Being Able to Communicate had the highest mean score (*M* = 2.93, SD = 1.36), followed by Not Being Able to Access Information (*M* = 2.83, SD = 0.97), Giving Up Convenience (*M* = 2.56, SD = 0.97), and Losing Connectedness (*M* = 2.12, SD = 0.95). The overall item‐level mean score for nomophobia was 2.62 (SD = 0.81) on the 5‐point response scale. Because the response format differed from the original 7‐point NMP‐Q and no validated severity thresholds were available for the adapted 5‐point version, the observed mean was not classified into mild, moderate, or severe nomophobia categories. As shown in Table 3, the 95% confidence intervals were relatively narrow, indicating precise estimates of the sample means.

**Table 3:** Item‐level descriptive statistics for nomophobia and its dimensions (*N* = 400).

Dimension	Mean (M)	SD	95% CI [lower, upper]
Not being able to access information	2.83	0.97	[2.74, 2.93]
Losing connectedness	2.12	0.95	[2.03, 2.21]
Not being able to communicate	2.93	1.36	[2.80, 3.07]
Giving up convenience	2.56	0.97	[2.46, 2.65]
Total nomophobia	**2.62**	**0.81**	**[2.54, 2.70]**

*Note:* All values are item‐level mean scores based on the adapted 5‐point Likert‐type response scale ranging from 1 (never) to 5 (always). Higher scores indicate greater nomophobia. Because validated severity thresholds were not available for the adapted 5‐point version, the scores were not classified into severity categories.

### 3.3 Relationships Among the Dimensions of Nomophobia

Pearson correlation analysis was conducted to examine the relationships among the four dimensions of nomophobia. As shown in Table 4, all dimensions were positively and significantly correlated with one another (all *p* < 0.001). The strongest association was observed between Not Being Able to Communicate and Losing Connectedness (*r* = 0.647), indicating that adolescents who experienced greater concerns about being unable to communicate also reported stronger feelings of losing connectedness. The weakest, although still moderate, association was found between Not Being Able to Communicate and Not Being Able to Access Information (r = 0.388). Overall, these findings suggest that the four dimensions of nomophobia are positively interrelated while representing distinct aspects of the construct.

**Table 4:** Descriptive statistics, reliability, and correlations among nomophobia dimensions.

Variable	M	SD	α	ω	1	2	3	4
1. Not being able to communicate	2.93	1.16	0.884	0.885	—			
2. Losing connectedness	2.11	0.89	0.767	0.768	0.647***	—		
3. Not being able to access information	2.83	0.97	0.701	0.705	0.388***	0.424***	—	
4. Giving up convenience	2.56	0.97	0.686	0.692	0.511***	0.524***	0.516***	—

*Note:* N = 400. Pearson correlation coefficients are reported below the diagonal. α = Cronbach's alpha; ω = McDonald's omega. ***p < 0.001.

### 3.4 Differences in Nomophobia By Demographic Characteristics and Smartphone‐Use Patterns

Table 5 presents differences in nomophobia scores according to demographic characteristics and smartphone‐use patterns. Female participants reported significantly higher nomophobia scores than male participants, *t*(398) = −7.89, *p* < 0.001, Cohen's d = 0.79. The mean difference between female and male participants was 0.59 points, 95% CI [0.44, 0.74]. Because measurement invariance of the adapted NMP‐Q across gender was not examined, this result represents a difference in observed scores and should not be interpreted as evidence of an invariant latent mean difference. No statistically significant differences were observed according to age, F(4, 395) = 0.62, *p* = 0.645, ηp^2^ = 0.006; grade level, F(4, 395) = 1.65, *p* = 0.160, ηp^2^ = 0.016; or parental attitudes toward internet and social media use, F(3, 396) = 1.47, *p* = 0.223, ηp^2^ = 0.011. Because Levene's test indicated unequal variances for age and grade level, supplementary Welch ANOVAs were conducted as sensitivity analyses. These analyses yielded the same substantive conclusions: neither age, Welch's F(4, 176.67) = 0.67, *p* = 0.614, nor grade level, Welch's F(4, 111.77) = 2.37, *p* = 0.057, was statistically significant. Homogeneity of variance was supported for the remaining comparisons, including daily smartphone use (all *p* > 0.05). The omnibus ANOVA was statistically significant for years of smartphone use, F(3, 396) = 2.90, *p* = 0.035, ηp^2^ = 0.021, and daily smartphone‐use duration, F(3, 396) = 7.56, *p* < 0.001, ηp^2^ = 0.054. However, none of the Tukey‐adjusted pairwise comparisons for years of smartphone use reached statistical significance. Accordingly, the significant omnibus result for years of smartphone use should not be interpreted as evidence that any specific ownership‐duration group differed significantly from another. Regarding daily smartphone use, participants who used smartphones for 4–6 h and 7 h or more per day reported significantly higher nomophobia scores than those who used smartphones for 1–3 h per day, both *p* < 0.01.

**Table 5:** Group differences in nomophobia scores by demographic characteristics and smartphone‐use patterns (*N* = 400).

Variable	Group	n	M	SD	Test statistic	df	p	Effect size
Gender	Male	196	2.32	0.73	t = −7.89	398	< 0.001	d = 0.79
	Female	204	2.91	0.77				
Age	13 years	99	2.59	0.81	F = 0.62	4, 395	0.645	ηp^2^ = 0.006
	14 years	87	2.61	0.90				
	15 years	46	2.48	0.78				
	16 years	91	2.67	0.82				
	17 years	77	2.69	0.69				
Grade	7	163	2.61	0.84	F = 1.65	4, 395	0.160	ηp^2^ = 0.016
	8	26	2.52	0.88				
	9	45	2.66	0.95				
	10	92	2.50	0.73				
	11	74	2.81	0.68				
Years of smartphone use	≤ 1 year	22	2.54	0.73	F = 2.90	3, 396	0.035	ηp^2^ = 0.021
	2–3 years	53	2.45	0.84				
	4–5 years	101	2.49	0.79				
	More than 5 years	224	2.72	0.81				
Daily smartphone use	< 1 h	13	2.55	0.73	F = 7.56	3, 396	< 0.001	ηp^2^ = 0.054
	1–3 h	114	2.33	0.81				
	4–6 h	166	2.69	0.82				
	≥ 7 h	107	2.81	0.72				
Parental attitude	Active monitoring	39	2.73	0.86	F = 1.47	3, 396	0.223	ηp^2^ = 0.011
	Warn but set no limits	239	2.66	0.75				
	No intervention	90	2.49	0.89				
	Never discuss usage	32	2.52	0.90				

*Note:* Mean scores were calculated using the adapted 5‐point Likert‐type response scale ranging from 1 (never) to 5 (always). Higher scores indicate greater nomophobia. Tukey HSD post hoc tests were conducted for statistically significant omnibus ANOVA results. For years of smartphone use, none of the pairwise comparisons reached statistical significance (all *p* > 0.05). For daily smartphone use, participants who used smartphones for 4–6 h and 7 h or more per day had significantly higher nomophobia scores than those who used smartphones for 1–3 h per day (both *p* < 0.01). M = mean; SD = standard deviation; t = independent‐samples t test; F = one‐way ANOVA. d = Cohen's d; ηp^2^ = partial eta squared. The 95% confidence interval for the gender mean difference is reported in the text.

### 3.5 Hierarchical Multiple Regression Analysis of Factors Associated with Nomophobia

A hierarchical multiple regression analysis was conducted to examine how gender, age, years of smartphone use, daily smartphone use, and parental attitudes were associated with adolescents' overall nomophobia scores. As shown in Table 6, gender and age were entered simultaneously in the first step. This model was statistically significant and accounted for 13.7% of the variance in nomophobia, R^2^ = 0.137, adjusted R^2^ = 0.133, F(2, 397) = 31.537, *p* < 0.001. Female adolescents reported significantly higher nomophobia scores than males (β = 0.368, *p* < 0.001), whereas age was not significantly associated with nomophobia (β = 0.045, *p* = 0.340).

**Table 6:** Hierarchical multiple regression of factors associated with nomophobia.

Predictor	Model 1				Model 2				Model 3			
	B	SE	β	p	B	SE	β	p	B	SE	β	p
Constant	1.954	0.382	—	< 0.001	2.302	0.408	—	< 0.001	2.615	0.413	—	< 0.001
Female	0.594	0.075	0.368	< 0.001	0.581	0.075	0.360	< 0.001	0.545	0.076	0.337	< 0.001
Age	0.024	0.025	0.045	0.340	0.007	0.026	0.013	0.792	−0.010	0.027	−0.019	0.706
Years of smartphone use												
≤ 1 year	—	—	—	—	−0.155	0.169	−0.044	0.360	−0.052	0.171	−0.015	0.761
2–3 years	—	—	—	—	−0.180	0.118	−0.076	0.127	−0.120	0.118	−0.050	0.310
4–5 years	—	—	—	—	−0.203	0.092	−0.109	0.028*	−0.172	0.093	−0.093	0.064
Daily smartphone use												
< 1 h	—	—	—	—	—	—	—	—	0.046	0.220	0.010	0.835
1–3 h	—	—	—	—	—	—	—	—	−0.300	.095	−0.168	0.002
≥ 7 h	—	—	—	—	—	—	—	—	0.129	0.093	0.071	0.167
Parental attitudes												
Active monitoring	—	—	—	—	—	—	—	—	0.216	0.130	0.079	0.098
No intervention	—	—	—	—	—	—	—	—	−0.145	0.093	−0.075	0.122
Never discuss usage	—	—	—	—	—	—	—	—	0.000	0.142	0.000	0.997
R^2^	0.137				0.150				0.193			
Adjusted R^2^	0.133				0.139				0.170			
ΔR^2^	0.137				0.013				0.043			
F	F(2, 397) = 31.537***				F(5, 394) = 13.894***				F(11, 388) = 8.456***			
ΔF	ΔF(2, 397) = 31.537***				ΔF(3, 394) = 1.977				ΔF(6, 388) = 3.485**			
Durbin–Watson	—				—				1.929			

*Note:* Dependent variable = nomophobia. Age was entered as a continuous variable in years. Reference categories were male, more than 5 years of smartphone use, 4–6 h of daily smartphone use, and parents who warned but did not restrict internet and social media use. B = unstandardized coefficient; SE = standard error; β = standardized coefficient. *p < 0.05. **p < 0.01. ***p < 0.001.

In the second step, years of smartphone use was added to the model. This produced a small and statistically nonsignificant increase in explained variance, ΔR^2^ = .013, ΔF(3, 394) = 1.977, *p* = 0.117. At this step, adolescents who had used smartphones for 4–5 years reported lower nomophobia scores than those who had used smartphones for more than 5 years (β = −0.109, *p* = 0.028). However, this association did not remain statistically significant after daily smartphone use and parental attitudes were included in the final model.

In the final step, daily smartphone use and parental attitudes were entered into the regression model. The final model explained 19.3% of the variance in nomophobia, R^2^ = 0.193, adjusted R^2^ = 0.170, ΔR^2^ = 0.043, ΔF(6, 388) = 3.485, *p* = 0.002, and was statistically significant, F(11, 388) = 8.456, *p* < 0.001. Gender remained positively associated with nomophobia (B = 0.545, SE = 0.076, 95% CI [0.396, 0.694], β = 0.337, *p* < 0.001), whereas age was not significantly associated with nomophobia (β = −0.019, *p* = 0.706). Among the newly added variables, only adolescents who used smartphones for 1–3 h per day reported significantly lower nomophobia scores than those using smartphones for 4–6 h per day, which constituted the reference category (B = −0.300, SE = 0.095, 95% CI [−0.486, −0.114], β = −0.168, *p* = 0.002). Neither years of smartphone use nor parental attitudes were significantly associated with nomophobia in the final model (*p* > 0.05). The Durbin–Watson statistic of 1.929 indicated that the assumption of independent residuals was satisfied.

A sensitivity analysis was conducted by combining participants who reported using smartphones for less than 1 h and those reporting 1–3 h per day into a single 3‐h‐or‐less category (n = 127), while retaining the 4–6‐h group as the reference category. The full sensitivity‐model results are presented in Supporting Information: Table S4. The model was statistically significant, F(10, 389) = 9.02, *p* < 0.001, and explained 18.8% of the variance in nomophobia (R^2^ = 0.188, adjusted R^2^ = 0.167). Compared with the 4–6‐h reference group, the 3‐h‐or‐less group reported significantly lower nomophobia scores (B = −0.268, SE = 0.093, 95% CI [−0.450, −0.086], β = −0.154, *p* = 0.004). Female gender also remained significantly associated with nomophobia (B = 0.543, SE = 0.076, 95% CI [.393, 0.692], β = 0.336, *p* < 0.001). The remaining predictors were not statistically significant. The results were consistent with the primary analysis in showing lower nomophobia scores among adolescents reporting 3 h or less of daily smartphone use than among those reporting 4–6 h.

As illustrated in Figure 1, female gender was positively associated with nomophobia, whereas using a smartphone for 1–3 h per day was negatively associated with nomophobia relative to the 4–6‐h reference category. The confidence intervals for the remaining predictors, including age, crossed zero.

**Figure 1:**
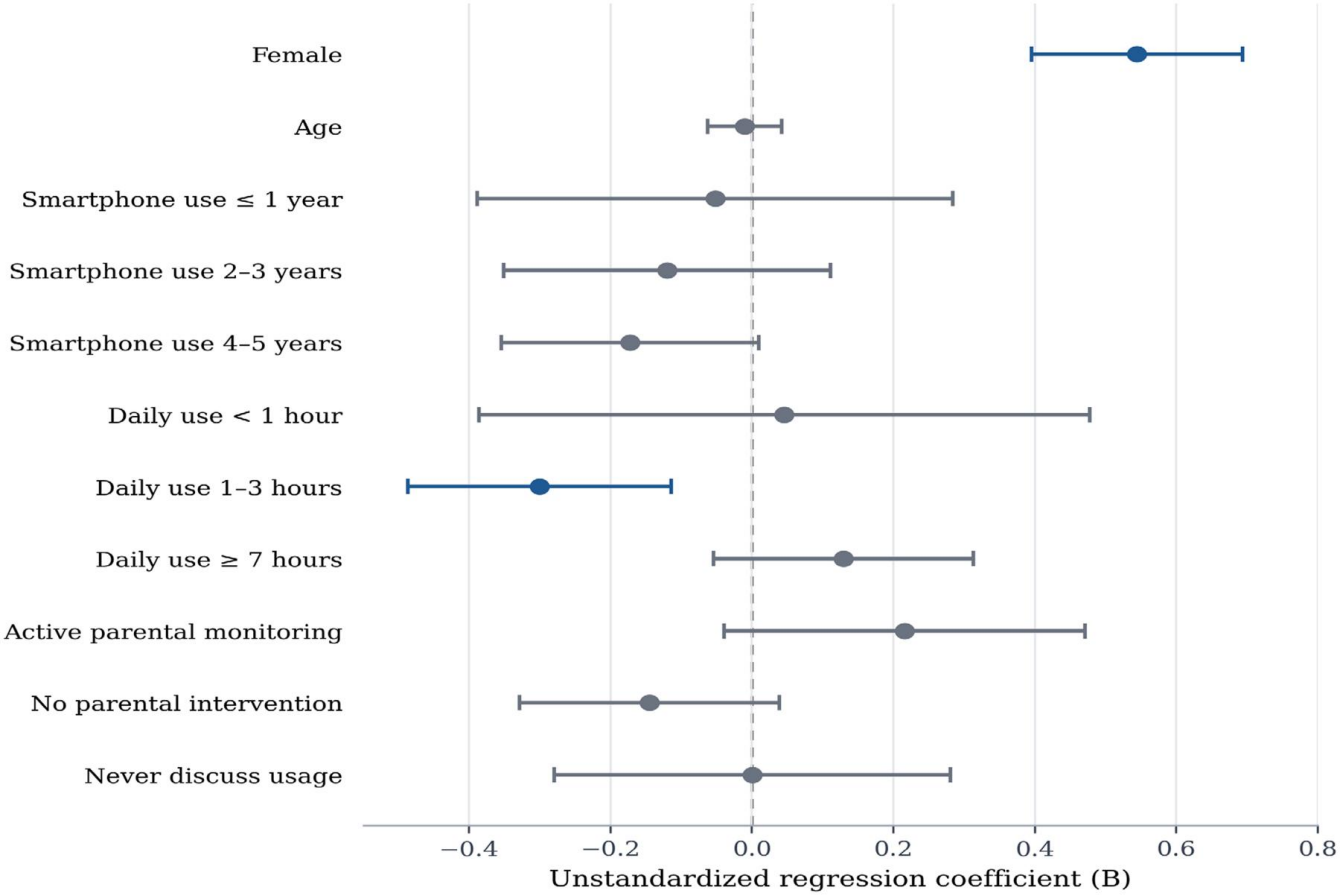
Unstandardized regression coefficients and 95% confidence intervals for the final regression model of nomophobia. Points represent unstandardized regression coefficients (B), and horizontal lines represent 95% confidence intervals. The vertical dashed line indicates no association (B = 0). Coefficients whose 95% confidence intervals do not include zero are statistically significant at *p* < 0.05. Reference categories were male, more than 5 years of smartphone use, 4–6 h of daily smartphone use, and parents who warned but did not restrict internet and social media use.

## 4 Discussion

This study extends the nomophobia literature by providing evidence from adolescents in Kyrgyzstan, an underrepresented population within Central Asian digital‐health research. The findings indicate that communication‐related concerns represented the dimension with the highest observed mean of nomophobia, while female gender and daily smartphone‐use patterns showed the most consistent associations with overall nomophobia scores. In contrast, age, grade level, parental attitudes, and years of smartphone use showed limited or inconsistent associations. These findings should be interpreted as context‐specific associations rather than causal effects or evidence of culturally determined smartphone behavior, given the cross‐sectional design and the absence of direct measures of cultural orientation.

The descriptive findings indicated an overall item‐level nomophobia score of 2.62 on the adapted 5‐point response scale. Because the original NMP‐Q severity thresholds were developed for the 7‐point version of the instrument, the observed mean was not classified into a specific severity category. Instead, the findings should be interpreted descriptively by comparing the observed mean scores of the four nomophobia dimensions. Among these dimensions, Not Being Able to Communicate had the highest mean score, whereas Losing Connectedness had the lowest.

This finding is consistent with the multidimensional conceptualization of nomophobia proposed by Yildirim and Correia [2], in which anxiety related to being unable to communicate represents one of the core dimensions of the construct. Adolescence is a developmental period characterized by increasing needs for peer interaction, social belonging, and interpersonal accessibility [29]. Consequently, interruptions in smartphone‐mediated communication may be perceived as particularly distressing during this stage of development. Previous evidence has also shown that communication disruption itself can contribute to communication‐related anxiety by limiting opportunities for social interaction and access to information [30]. Although that evidence was obtained in a disaster context, it supports the broader interpretation that maintaining uninterrupted communication has important psychological value. Consistent with this interpretation, the strong positive association observed between *Not Being Able to Communicate* and *Losing Connectedness* suggests that concerns about communication interruption are closely linked to adolescents' perceptions of social disconnection.

Social Cognitive Theory provides a useful framework for interpreting why communication‐related concerns may become salient in adolescents' smartphone use [10]. From this perspective, behavioral patterns and psychological responses develop through reciprocal interactions among personal characteristics, behavior, and environmental influences. Repeated exposure to notifications, peer responses, and expectations of continuous availability may reinforce habitual smartphone engagement and increase sensitivity to communication interruption. However, reinforcement processes, habitual checking, and perceived expectations of availability were not directly measured in the present study. Social Cognitive Theory should therefore be viewed as an interpretive framework rather than as an empirically tested explanatory mechanism.

The findings indicate that, among the demographic variables included in the regression model, gender was significantly associated with nomophobia, whereas age was not. Female adolescents reported higher nomophobia scores than male adolescents, and gender showed the strongest association in the final regression model after controlling for age, smartphone‐use patterns, and parental attitudes. However, measurement invariance across gender was not tested for the adapted NMP‐Q. Accordingly, the observed difference should be interpreted cautiously, as it cannot be determined whether the scale functioned equivalently for female and male adolescents or whether some portion of the difference reflected gender‐related measurement effects.

The observed gender difference is broadly consistent with previous studies reporting higher nomophobia levels among female adolescents and young adults [14, 31]. One possible explanation is that female adolescents may use smartphones more intensively for interpersonal communication and social interaction, potentially increasing concerns about losing access to communication. However, the present study did not examine communication motives, social expectations, or emotional attachment to smartphone use directly. Therefore, these explanations remain speculative and should be investigated in future research. Moreover, although Arpaci [15] suggested that cultural context may influence patterns of digital attachment, the present study did not measure cultural values or collectivism directly. Consequently, the observed gender difference should be interpreted as an empirical finding rather than as evidence of a specific cultural mechanism.

The final hierarchical regression model explained 19.3% of the variance in adolescents' nomophobia scores. Although this represents a meaningful contribution for a model based primarily on demographic characteristics and self‐reported smartphone‐use patterns, most of the variance remained unexplained. Nomophobia is therefore unlikely to be explained adequately by usage duration and basic demographic variables alone. Previous research indicates that psychological and interpersonal factors—including loneliness, social anxiety, fear of missing out, problematic smartphone use, perceived social support, and difficulties in emotion regulation—may also be associated with nomophobia [1, 4, 31]. Future studies should test more comprehensive explanatory models incorporating these variables and examine their direct, mediating, or moderating roles.

Recent evidence from clinical adolescent and family samples further illustrates the potential relevance of psychological and contextual factors not assessed in the present study. Among children diagnosed with ADHD, overall nomophobia did not differ significantly from healthy controls and was not associated with anxiety or depression; however, the Giving Up Convenience dimension was significantly higher in the ADHD group, and girls with ADHD reported greater concerns related to Not Being Able to Communicate than boys [32]. Similarly, adolescents diagnosed with obsessive‐compulsive disorder exhibited higher problematic smartphone use than healthy controls, whereas overall nomophobia did not differ significantly between the groups; associations with impulsivity nevertheless suggested that individual psychological characteristics may contribute to smartphone‐related difficulties [33]. Evidence from a parental sample also indicated positive associations of nomophobia with anxiety and depressive symptoms, with depression showing an independent association with nomophobia [34]. Taken together, these studies suggest that psychological distress, impulsivity, clinical characteristics, and family context may help account for variance beyond smartphone‐use duration. However, findings from younger children, clinical adolescent groups, and adult parental samples cannot be considered directly equivalent to those obtained from the community‐based adolescent sample examined here and should therefore be regarded as hypothesis‐generating evidence.

Although the omnibus ANOVA indicated an overall difference across ownership‐duration groups, none of the Tukey‐adjusted pairwise comparisons reached statistical significance, and years of smartphone use was not significantly associated with nomophobia in the final regression model. Therefore, the omnibus result should not be interpreted as evidence that any particular ownership‐duration group had higher or lower nomophobia scores than another. This pattern suggests that simply owning a smartphone for a longer period may be less relevant to nomophobia than the intensity of current daily engagement. However, ownership duration may not accurately reflect the frequency, purpose, or emotional significance of smartphone use. Future studies should therefore assess not only how long adolescents have owned smartphones but also how they use them, including communication, social networking, entertainment, academic activities, and habitual checking.

In contrast, daily smartphone use demonstrated a clearer and more consistent association with nomophobia than years of smartphone ownership. Adolescents who reported using smartphones for 4–6 h or 7 h or more per day generally exhibited higher nomophobia scores than those reporting 1–3 h of daily use, although only the comparison with the 4–6‐h reference category remained statistically significant in the final regression model. This finding is consistent with previous research indicating that greater daily smartphone engagement is associated with higher levels of nomophobia and problematic smartphone‐related behaviors [1, 7, 35]. One possible explanation is that frequent smartphone use may reinforce expectations of constant accessibility and immediate communication, thereby increasing discomfort when mobile access is interrupted. At the same time, the reverse relationship is also plausible, whereby adolescents with higher nomophobia may use their smartphones more frequently to reduce anxiety associated with disconnection. Because the present study employed a cross‐sectional design, the direction of this relationship cannot be determined. Longitudinal studies are therefore needed to clarify whether intensive smartphone use contributes to the development of nomophobia or whether pre‐existing nomophobia promotes more frequent smartphone use.

Parental attitudes toward internet and social media use were not significantly associated with adolescents' nomophobia and did not contribute significantly to the final regression model after gender and smartphone‐use patterns were considered. This result should not necessarily be interpreted as indicating that parents have no influence on adolescents' digital behavior. Rather, the broad response categories used in the present study may not have captured the multidimensional nature of parental mediation. Contemporary research distinguishes among active discussion, restrictive mediation, monitoring, co‐use, and technical control, and these strategies may differ in both their frequency and their effects on adolescents' smartphone‐related behavior [36, 37]. Recent evidence also suggests that the relationship between parental mediation and problematic smartphone use may depend on underlying family processes and the specific mediation strategy employed rather than on the mere presence of parental guidance [38]. In the present sample, parental guidance appeared to consist primarily of verbal warnings without formal restrictions, which may reflect active guidance but cannot be interpreted confidently because the frequency, consistency, and quality of parent–adolescent communication were not measured. Accordingly, the nonsignificant result may reflect limited measurement sensitivity rather than the absence of a parental role.

Future studies should therefore employ validated multidimensional parental‐mediation measures that distinguish active mediation, restrictive mediation, monitoring, co‐use, technical control, and the quality of parent–adolescent communication. Such measures may clarify whether particular parental strategies are associated with lower or higher levels of nomophobia and whether these associations vary according to adolescents' age, gender, and intensity of smartphone use. Cross‐cultural comparisons would also be valuable for examining whether parental mediation operates differently across social settings. However, because the present study did not directly assess cultural values, collectivism, family communication quality, or specific mediation strategies, the nonsignificant parental‐attitude findings should not be attributed to a particular cultural mechanism. Cultural context should therefore be regarded as background for interpreting the findings rather than as an explanation directly tested in the study.

Taken together, the findings support a multidimensional and context‐sensitive understanding of adolescent nomophobia. From a practical perspective, the findings may help identify potential areas for the future development of adolescent digital well‐being initiatives, including balanced communication habits, digital well‐being education, and awareness of intensive daily smartphone use.

However, because no intervention was evaluated, these suggestions should be regarded as possible directions for program development and future testing rather than as evidence of intervention effectiveness.

## 5 Conclusion

This study provides preliminary, context‐specific evidence on nomophobia among 400 adolescents recruited through convenience sampling in Bishkek. Communication‐related concerns represented the dimension with the highest observed mean of nomophobia. Gender showed the strongest association with observed nomophobia scores; however, because measurement invariance was not tested, the observed female–male score difference cannot be interpreted as a confirmed difference in latent nomophobia. In the final regression model, adolescents reporting 1–3 h of daily smartphone use had lower observed nomophobia scores than those reporting 4–6 h, the reference category; age, years of smartphone use, and parental attitudes showed limited or nonsignificant associations. The final model accounted for 19.3% of the variance, indicating that additional psychological, interpersonal, and family‐related factors remain to be examined.

Because the study was cross‐sectional and based on a single urban convenience sample, the findings should not be interpreted causally or generalized to all adolescents in Kyrgyzstan. The psychometric evidence for the adapted five‐point NMP‐Q should also be regarded as preliminary. Future research should independently validate the instrument and use larger, more diverse, and representative samples drawn from different regions, including both urban and rural settings. Despite these limitations, the study provides an initial basis for understanding adolescent nomophobia within an underrepresented Central Asian context.

## 6 Limitations

This study has several limitations that should be considered when interpreting the findings. First, participants were recruited at the individual level through a family‐mediated convenience‐sampling procedure based on the researchers' personal and local community contacts in central Bishkek. Schools were not formally selected as recruitment sites, and participants' school names or affiliations were not recorded. Consequently, the number and distribution of schools represented in the sample could not be determined, and possible clustering or school‐level selection effects could not be examined. The sample may therefore not represent adolescents attending private schools, living in rural areas or other regions of Kyrgyzstan, or experiencing different socioeconomic and digital‐access conditions. In addition, 50 of the 450 distributed questionnaires were either not returned or contained substantial missing responses. Because the characteristics of these adolescents could not be compared with those included in the final sample, nonresponse bias cannot be ruled out. The findings should therefore be generalized beyond the present urban, individually recruited convenience sample with caution.

Second, the study relied on self‐report measures, which may be subject to recall and social‐desirability biases. In addition, because questionnaire forms were distributed and returned through parents, some adolescents may have perceived that their responses could be seen by a parent. This possibility may have influenced responses to questions concerning parental mediation, smartphone use, or social media behavior, even though no directly identifying information was collected. Although parents were asked not to assist, influence, or intervene while adolescents completed the questionnaires, no sealed‐return procedure was used, and parental access to completed responses could not be fully excluded. Future studies should use privacy‐enhancing procedures, such as sealed return envelopes or direct researcher‐administered data collection, and could incorporate observational data or objective digital‐use records to improve measurement accuracy.

Third, the cross‐sectional design restricts the ability to draw causal inferences. While the study identifies associations between variables, the directionality of these relationships cannot be determined. For example, greater daily smartphone use may contribute to higher nomophobia, but adolescents with higher nomophobia may also be more likely to use their smartphones for longer periods. Longitudinal designs would provide a clearer understanding of how nomophobia develops and changes over time.

Fourth, parental attitudes toward internet and social media use were measured using broad categorical responses. Although this approach provided an overview of parental practices, it did not capture specific dimensions such as active mediation, restrictive mediation, monitoring, co‐use, or the quality of parent–child communication. Future studies should use multidimensional parental mediation measures to examine these processes in greater detail.

Fifth, the present study used a Russian‐language 5‐point adaptation of the NMP‐Q that differed from the original 7‐point response format. Although the five‐category format was considered more developmentally accessible for adolescents, the change in the number and wording of the response categories altered the original scoring metric, prevented the application of the original severity thresholds, and may limit direct comparability with studies using the original 7‐point version. Furthermore, the mixed CFA fit indices, particularly the CFI and TLI values below 0.90, indicate that the four‐factor structure was not strongly confirmed. Because the EFA and CFA were conducted using the same sample, the findings were not independently cross‐validated and should be regarded as preliminary. Measurement invariance across gender was also not examined. Given the mixed fit of the baseline four‐factor CFA model, configural, metric, and scalar invariance testing was not pursued. Consequently, the observed gender difference cannot be assumed to reflect fully equivalent measurement of the construct across female and male adolescents and should be interpreted cautiously. Although the present authors examined linguistic and conceptual equivalence through back‐translation and qualitatively reviewed the items for clarity and cultural suitability, a quantitative content‐validity index was not calculated. Future studies should independently validate the Russian‐language 5‐point version and assess its measurement equivalence with the original response format. Future validation studies should also consider ordinal‐data estimators, such as WLSMV, to examine the robustness of the factor structure.

Sixth, the regression model explained 19.3% of the variance in nomophobia, indicating that a substantial proportion of the variance remained unexplained. Psychological and interpersonal variables such as loneliness, social anxiety, fear of missing out, self‐control, emotion regulation, perceived social support, and problematic smartphone use were not examined in the present study. Including these factors in future research may provide a more comprehensive understanding of adolescent nomophobia.

Finally, the study focused exclusively on adolescents. Nomophobia may manifest differently across developmental stages and age groups. Comparative studies involving adolescents, young adults, and older populations could provide a more comprehensive understanding of how smartphone‐related anxiety varies across the lifespan.

Despite these limitations, the study contributes to the literature by providing empirical evidence from an underrepresented Central Asian context, documenting the relative importance of communication‐related concerns, gender, and daily smartphone use, and offering a context‐sensitive perspective on adolescent nomophobia.

## Author Contributions

**Maksutova Begaiym:** conceptualization, writing – review and editing. **Ergeshova Seyil Baktybekovna:** conceptualization, writing – original draft, data curation. **Bedir Eroğlu:** conceptualization, writing – original draft, methodology, writing – review and editing, data curation.

## Funding

The authors have nothing to report.

## Ethics Statement

Ethical approval for the study was obtained from the Ethics Committee of Osh State University, Kyrgyzstan (Protocol No. 6, June 1, 2026). Written informed consent was obtained from the parents or legal guardians of all participating adolescents, and written assent was obtained from the adolescents before participation. Participation was voluntary, and no directly identifying information was collected. All procedures were conducted in accordance with the Declaration of Helsinki.

## Conflicts of Interest

The authors declare no conflicts of interest.

## Transparency Statement

The corresponding author, affirms that this manuscript is an honest, accurate, and transparent account of the study being reported; that no important aspects of the study have been omitted; and that any discrepancies from the study as planned (and, if relevant, registered) have been explained.

## Supporting information


**Supporting Table S1:** Standardized Confirmatory Factor Analysis Loadings for the Adapted Five‐Point NMP‐Q.
**Supporting Table S2:** Standardized Correlations Among the Four Latent Nomophobia Factors.
**Supporting Table S3:** Rotated Pattern Matrix for the Four‐Factor EFA of the Russian‐Language 5‐Point NMP‐Q.
**Supporting Table S4:** Sensitivity Regression Model for Factors Associated with Nomophobia.

## Data Availability

The data used in this research are not publicly available due to privacy principles; however, they can be obtained from the corresponding author upon reasonable request.
