# Supplementary material for: Nomophobia Among Adolescents in Kyrgyzstan: Scores, Dimensions, and Associations With Smartphone‐Use Patterns
Source: Health Sci Rep. 2026 Nov 10;9(11):e73501. doi: 10.1002/hsr2.73501 (PMC13749418; doi:10.1002/hsr2.73501)
Supplement: Supplementary file 1 [file HSR2-9-e73501-s001.docx]

**Supplementary Material**

*Standardized CFA Loadings and Latent Factor Correlations for the Adapted Five-Point NMP-Q*

**Table S1**
*Standardized Confirmatory Factor Analysis Loadings for the Adapted Five-Point NMP-Q*

| Item | Factor | Standardized loading |
| --- | --- | --- |
| NF1 | Not Being Able to Access Information | .604 |
| NF2 | Not Being Able to Access Information | .649 |
| NF3 | Not Being Able to Access Information | .516 |
| NF4 | Not Being Able to Access Information | .677 |
| NF5 | Giving Up Convenience | .533 |
| NF6 | Giving Up Convenience | .607 |
| NF7 | Giving Up Convenience | .553 |
| NF8 | Giving Up Convenience | .549 |
| NF9 | Giving Up Convenience | .540 |
| NF10 | Not Being Able to Communicate | .705 |
| NF11 | Not Being Able to Communicate | .727 |
| NF12 | Not Being Able to Communicate | .836 |
| NF13 | Not Being Able to Communicate | .831 |
| NF14 | Not Being Able to Communicate | .701 |
| NF15 | Not Being Able to Communicate | .696 |
| NF16 | Losing Connectedness | .812 |
| NF17 | Losing Connectedness | .537 |
| NF18 | Losing Connectedness | .529 |
| NF19 | Losing Connectedness | .740 |
| NF20 | Losing Connectedness | .390 |

***Note.*** Values are fully standardized CFA loadings. Model parameters were estimated using maximum likelihood.

**Table S2**
*Standardized Correlations Among the Four Latent Nomophobia Factors*

| Factor | 1 | 2 | 3 | 4 |
| --- | --- | --- | --- | --- |
| 1. Not Being Able to Access Information | 1.000 |  |  |  |
| 2. Giving Up Convenience | .729 | 1.000 |  |  |
| 3. Not Being Able to Communicate | .488 | .644 | 1.000 |  |
| 4. Losing Connectedness | .542 | .714 | .840 | 1.000 |

*Note.* Values are standardized latent-factor correlations estimated using maximum likelihood. All correlations were statistically significant at p < .001.

**Supplementary Table S3**
*Rotated Pattern Matrix for the Four-Factor EFA of the Russian-Language 5-Point NMP-Q*

| Item | Factor 1 | Factor 2 | Factor 3 | Factor 4 |
| --- | --- | --- | --- | --- |
| NF1 | .61 | .04 | −.04 | .06 |
| NF2 | .61 | .05 | .00 | .03 |
| NF3 | .42 | .03 | .06 | .13 |
| NF4 | .61 | .06 | .05 | .01 |
| NF5 | .22 | .28 | .12 | .05 |
| NF6 | .10 | .66 | −.06 | .03 |
| NF7 | .05 | .46 | .10 | .11 |
| NF8 | .07 | .52 | .17 | −.14 |
| NF9 | .44 | .11 | .16 | −.02 |
| NF10 | .21 | −.05 | .54 | −.08 |
| NF11 | −.03 | .00 | .78 | −.08 |
| NF12 | .10 | .05 | .77 | −.03 |
| NF13 | .09 | .00 | .86 | −.11 |
| NF14 | −.17 | .09 | .69 | .14 |
| NF15 | .04 | −.13 | .61 | .31 |
| NF16 | −.09 | .16 | .65 | .24 |
| NF17 | .02 | .16 | .05 | .59 |
| NF18 | .06 | .21 | −.06 | .39 |
| NF19 | .15 | .16 | .35 | .30 |
| NF20 | .16 | −.12 | .01 | .59 |

**Note.** Extraction method = principal axis factoring. Rotation method = Direct Oblimin. All pattern loadings across the four factors are reported; no loading-suppression threshold was applied.

**Supplementary Table S4**
*Sensitivity Regression Model for Factors Associated with Nomophobia*

| Predictor | B | SE | β | 95% CI for B | p |
| --- | --- | --- | --- | --- | --- |
| Constant | 2.633 | .414 | — | [1.820, 3.447] | < .001 |
| Female | .543 | .076 | .336 | [.393, .692] | < .001 |
| Age | −.012 | .027 | −.022 | [−.065, .041] | .658 |
| Years of smartphone use: ≤1 year | −.022 | .170 | −.006 | [−.357, .313] | .897 |
| Years of smartphone use: 2–3 years | −.116 | .118 | −.049 | [−.348, .115] | .323 |
| Years of smartphone use: 4–5 years | −.168 | .093 | −.090 | [−.351, .015] | .071 |
| Daily smartphone use: ≤3 hours | −.268 | .093 | −.154 | [−.450, −.086] | .004 |
| Daily smartphone use: ≥7 hours | .131 | .094 | .072 | [−.053, .315] | .163 |
| Parental attitude: Active monitoring | .211 | .130 | .077 | [−.045, .466] | .106 |
| Parental attitude: No intervention | −.128 | .093 | −.066 | [−.311, .054] | .168 |
| Parental attitude: Never discuss usage | .028 | .142 | .009 | [−.251, .306] | .846 |

**Note.** *N* = 400. Dependent variable = nomophobia. Reference categories were male, more than 5 years of smartphone use, 4–6 hours of daily smartphone use, and parents who warned adolescents about online risks but did not restrict internet or social media use. *B* = unstandardized coefficient; *SE* = standard error; β = standardized coefficient; CI = confidence interval.
